# Digital Access in Working-Age and Older Adults and Their Caregivers Attending Psychiatry Outpatient Clinics: Quantitative Survey

**DOI:** 10.2196/aging.9130

**Published:** 2018-11-22

**Authors:** Callum Cruickshank, Donald J MacIntyre

**Affiliations:** 1 Division of Psychiatry Centre for Clinical Brain Sciences University of Edinburgh Edinburgh United Kingdom

**Keywords:** general psychiatry, internet access, memory assessment and treatment service, mobile phone, old age psychiatry

## Abstract

**Background:**

It has been suggested that improving access to mental health services, supporting self-management, and increasing clinical productivity can be achieved through the delivery of technology-enabled care by personal mobile-based and internet-based services. There is little evidence available about whether working-age and older adults with mental health problems or their caregivers have access to these technologies or their confidence with these technologies.

**Objective:**

This study aimed to ascertain the prevalence and range of devices used to access the internet in patients and caregivers attending general and older adult psychiatry outpatient services and their confidence in using these technologies.

**Methods:**

We conducted an anonymous survey of 77 patients and caregivers from a general psychiatry and old age psychiatry clinic to determine rates of internet access and device ownership, and attitudes to technology-enabled care.

**Results:**

We found high levels of internet access and confidence in using the internet in working-age adults, their caregivers, and older adult caregivers but not in older adult patients. The smartphone usage predominated in working-age adults and their caregivers. Older adult caregivers were more likely to use desktop or laptop computers. In our sample, tablets were the least popular form factor.

**Conclusions:**

Access rates and uptake of internet-based services have the potential to be high in working-age adults and their caregivers but are likely to be markedly lower among older adult patients attending psychiatry clinics. Applications designed for tablets are likely to have low uptake. All groups identified appointment reminders as likely to be beneficial.

## Introduction

It is recognized that although the majority of people with mental health problems never seek help [1], current United Kingdom (UK) services are struggling with rising demand in a time of severely constricted resources [2]. One strategy that seeks to address these dilemmas is to improve access to mental health services, support self-management, and increase clinical productivity, through the adoption of technology-enabled care [3].

The launch of iPhone in 2007, Android handsets in 2008, followed by Apple’s iPad tablet computers in 2010, combined with the increasing prevalence of Wi-Fi and mobile broadband, have led to the widespread public adoption of powerful mobile technology. Smartphones overtook all other computing devices in popularity in the UK in 2015. As many as 90% of young adults now own a smartphone, and adults use a smartphone for nearly 2 hours a day on average.

In older adults, the situation is more complex; in the United Kingdom, 3 in 10 adults aged 65-74 years, two-thirds of those aged ≥75 years, and a quarter of those in the lowest socioeconomic group of older adults do not use the internet at all [4]. In 2015, a large US study [5] of 3116 adults aged >65 years found that only 15.1% had access to the internet through a handheld device. Among those aged >65 years, the youngest, wealthiest, and best-educated participants were most likely to have internet access, but the study did not describe the mental health status of participants. Given that many people living with mental health problems are older, and suffer socioeconomic disadvantage [6] it is a concern that digital exclusion could mean that ambitions to revolutionize mental health care through technology may fall at the first hurdle.

Given the lack of available data on internet access among people with mental illness and that estimates of technology use that do exist vary markedly and do not tend to include considerations of internet access [7], we decided to survey our general psychiatry clinic (which included a mix of patients aged from 18 to 65 years and caregivers attending for psychological therapy, planned and emergency psychiatric assessment and follow-up, and a clozapine phlebotomy service) and a separate specialist older adults (aged >65 years) memory clinic. We compare our findings to general population data collected by Ofcom [4] and explore the implications.

## Methods

Our project was reviewed by the local Ethics Committee, which determined that full ethical approval was not required. The study was approved by the Royal Edinburgh Hospital Quality Improvement Team. An anonymous survey (see Multimedia Appendix 1) was conducted by inviting all those attending the Royal Edinburgh Hospital Outpatient Department and the Memory Assessment and Treatment Service (MATS) from April 8, 2016 to May 6, 2016. Both patients and their caregivers were surveyed. Respondents were asked whether the appointment they were attending was for themselves or for someone they were accompanying. The survey was administered by clerical staff at each outpatient clinic and was also available on tables in the waiting rooms with posters inviting patients to participate. One of the authors (CC) visited the general psychiatry outpatient clinic and directly brought the survey to the attention of patients. Survey questions were written in simple English, taking care to avoid technical jargon, using a mix of dichotomous questions, free response questions, and 5-point Likert scales. This survey was designed to be short and easy to understand, especially for people with dementia. Moreover, exact wording was chosen with reference to questions in adults’ media use and attitudes to ensure responses were comparable.

## Results

In the general psychiatry outpatient clinic, there were 11 male and 13 female patient attendees whose ages spanned from 18 and >75 years and 6 male and 12 female caregiver attendees whose ages spanned from 24 and 74 years. In the MATS clinic, there were 8 male and 5 female patient attendees aged >75 years and 9 male and 13 female caregiver attendees whose ages spanned from 18 and >75 years.

The overall prevalence of internet access was high, with 85% (63/74) respondents reporting access to the internet. Of note, 95% (35/37) of caregivers and 75% (28/36) of patients had access to the internet. However, only 54% (7/13) of the patient group attending MATS had any internet access, compared with 88% (21/24) of the general psychiatry group (general psychiatry outpatient clinic). Of those with the internet, the majority had an internet connection in their home, and the remaining 2 had access at public libraries.

In addition, the mobile phone ownership was high, with 90% (66/73) respondents owning a mobile phone. Notably, 96% (22/23) of general psychiatry patients and 70% (9/13) of MATS patients owned a mobile phone. Similarly, all general psychiatry caregivers (n=18) had mobile phones, while 89% (17/18) of MATS caregivers did. Most respondents were able to access the internet on their phones; however, 27% (20/74) were not able to because they did not own a phone or because their phone could not connect to the internet. This was true for 77% (10/13) of MATS patients and 21% (4/19) of MATS caregivers, but only for 6% (1/18) of general psychiatry caregivers and 21% (4/19) of general psychiatry patients.

Mobile phones were the most popular device in all groups, other than MATS caregivers, where personal computers or laptops were ubiquitous (n=19). Even in that group, 90% (17/19) had a mobile phone in the household, and the same number had a tablet. Overall though, tablets were the least popular device with fewer than half of general psychiatry patients (11/24) and caregivers (8/18) and MATS patients (6/13) having access to one. For general psychiatry patients and caregivers and MATS patients, personal computers and laptops were available to 75% (18/24), 78% (14/18), and 62% (8/13), respectively.

Caregivers were more confident using the internet than patients, and the general psychiatry group tended to be more confident than the MATS group.

**Figure 1:**
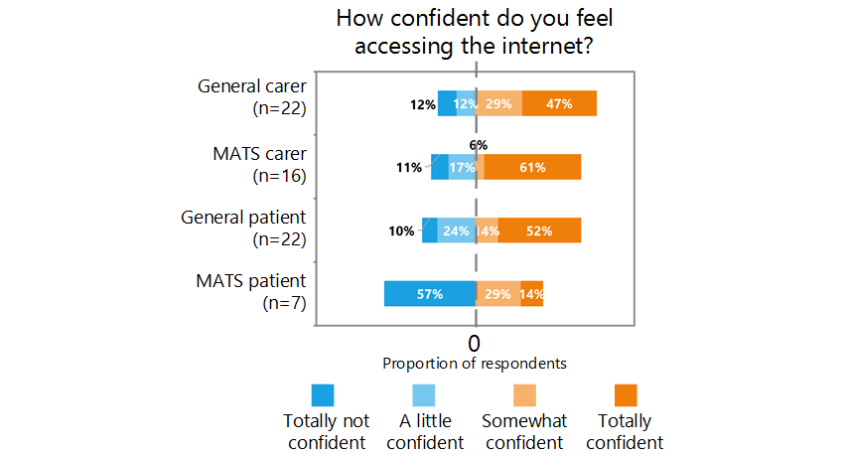
Respondents’ ratings on a Likert scale regarding their confidence in accessing the internet. MATS: memory assessment and treatment service.

Within the MATS group, 56% (10/18) of respondents had a mobile phone, but only 22% (4/18) of respondents had a phone that could connect to the internet. The overall internet access was higher with 50% (9/18) reporting access to the internet. Only 11 of those aged >75 years rated their confidence with accessing the internet, but 6 stated they were totally not confident, 2 a little confident, and 3 totally confident. This limited sample suggests they do not feel confident accessing the internet. Furthermore, personal computer and tablet ownership was similar to that of mobile phones among those aged >75 years in the MATS group with 10 and 8 of 18 respondents, respectively, owning those devices. Figure 1 shows the ratings provided by the respondents on a Likert scale regarding their confidence in accessing the internet. No respondent answered “neither,” and no MATS patient responded “A little confident.” These values are not shown.

Several patients commented on the survey as follows:

Fine for younger caregivers but no use for older patients. There could be no confidence that messages, etc, would be picked up.

MATS Caregiver, 55-64

Father would be completely unable to use technology, but would think that he could.

MATS patient, 75+ (completed on behalf of father)

A vital part of being kept informed. Mobile phone iPad PC emails.

MATS patient, 75+

Appointment reminders via email/text would be very useful

18-24, general psychiatry patient

## Discussion

### Principal Findings

The overall finding of this paper is encouraging—(63/74) 85% of patients and caregivers attending psychiatric clinics in Edinburgh reported that they had access to the internet; overall, (61/74) 82% had access to the internet at home. This is broadly similar to the UK general population rate of 86% and 84%, respectively.

### Older Adult Clinic

At our older adults’ clinic (MATS), in the patient group, 46% (6/13) did not have access to the internet; in this group, all were all aged over 75 years, while the caregiver group at the older adults clinic was aged from 18-24 to >75 years. Our findings are in line with recent UK general population surveys, which report that the >75 age group is the least likely to have internet access, and up to two-thirds had no access at all [4].

In our sample, MATS patients were also the least likely to be able to access the internet on their phone (3/13), suggesting that they would not be able to access the internet in the waiting room or a clinic. Our sample is again in line with UK population estimates, which report that only 18% of those aged 65-74 years and 4% of those aged >75 years accessed the internet through a smartphone [4], and with Shahrokni et al [5] who found that 15.6% of those aged >65 years in their survey of 3116 people had accessed the internet through a handheld device. In addition, they found that the group that had accessed the internet using a handheld device comprised the youngest, wealthiest, and best-educated participants. This is of great concern given that people with mental health conditions are likely to belong to lower socioeconomic groups [6] and people with dementia are likely to be older. This may place patients with dementia as among the least likely to access the internet through handheld devices.

Ofcom [4] found that those aged 65-74 years and >75 years used tablets more often than smartphones, but this only accounted for 23% and 13%, respectively, suggesting that services developed for tablets may not be currently able to best address the needs of older adults; this may impact the types of services they are able to access. If older adults are to use internet-based services, they may have to be directly targeted at this group to overcome these particular barriers, something which is not happening at present [8].

### General (Working Age) Clinic

In a survey of 100 patients at a general psychiatry outpatient clinic in the United Kingdom, Glick et al [8] found that while 85% of patients with serious mental illness had a mobile phone, only 37% owned a smartphone. Firth et al [9] conducted a meta-analysis and found the rates to be 81.4% and 35%. However, in our general psychiatry sample, (22/23) 96% were found to own a mobile phone, and (19/23) 83% had internet-enabled phones. Tablet computers were the least popular computing device in our general sample, although 57% (42/74) had access to a tablet. In sharp contrast to the older adult clinics, rates of internet access here are high, suggesting that internet-dependent services may be more widely used within this group.

### Socioeconomic and Geographic Factors

Internet access rates vary with the socioeconomic status and are likely to be lower in more rural areas. Handley et al surveyed 1246 patients in rural Australia in 2014 to determine the feasibility of internet-based mental health treatments using 2 feasibility criteria—(1) internet access and (2) willingness to use internet services. This Australian study found that only 7% of those who would consider using an internet service cited lack of internet access as a barrier [10], but that rates of internet access decreased markedly with increasing rurality, which is also true in Scotland [11].

### Qualitative Feedback: Attitudinal Factors

The second barrier to uptake identified by Handley et al was the willingness to use internet-based services. As well as having poorer internet access, older adult clinic attendees may be the less likely to see value in using digital services. Qualitative feedback in our survey, which prompted “If there’s anything you’d like to say about the use of technology in care, please do so here,” included remarks such as:

Fine for younger caregivers but no use for older patients. There could be no confidence that messages, etc, would be picked up.

MATS Caregiver, 55-64

It is recognized that if users are unwilling or simply uninterested in using digital technology, then they will not integrate it into their daily routines [12]. Conversely, we also found concern expressed that older adult patients may overestimate their capability:

Father would be completely unable to use technology, but would think that he could

Caregiver of patient aged 75+

Sourbati [12] found that, in general, older people had very little idea how internet-based services might benefit them.

Clearly, health care needs to be tailored to the needs of an individual. One older adult commented:

A vital part of being kept informed. Mobile phone iPad PC emails.

MATS patient, 75+

In general, we found younger patients and caregivers were more enthusiastic about the benefits of internet-based services and identified clear, practical benefits, such as appointment reminders and the provision of additional information, as beneficial. In addition, Firth et al [9] found appointment reminders and enhanced communication with health services to be the most sought after use for technology. Moreover, respondents in our study valued digital communication with our services. However, a recurring theme is service users’ concern that implementing technology may be used as an excuse to reduce services and, in particular, face-to-face contact [13,14], although some studies suggest social benefits [15].

### Trust and Equity

Another recurring theme in digital health care is trust. Despite their abundance, the regulation of digital health apps is still being developed [16]. Given the potential pitfalls of misinformation and poor health advice, it seems appropriate that health care services should have a role in identifying and curating technology-enabled care. Patients in this sample identified a difference between apps and “helping care,” suggesting a lack of trust:

I have no knowledge of what “technology in care” is—is it helping care through technology or is it apps?

35-44, general psychiatry caregiver

At present, there is conflicting advice on apps for mental health. Bennion et al [7] collected data on internet-based services used to treat or manage stress, anxiety, or depression in English health trusts using freedom of information requests, finding 41 different apps or services recommended by the National Health Service (NHS) in England. They highlighted the lack of uniformity in making these recommendations and that there were no apps designed for people over the age of 65 years.

Furthermore, in moving to greater use of digital services, barriers to adoption must be recognized and efforts made to ensure equitable access. Our study suggests that, at present, older adult patients and caregivers attending psychiatry clinics are less likely to use digital services, particularly if these are tablet or smartphone based; however, if these tools are personalized to an individual’s circumstances, uptake is still possible.

### Limitations

The principal limitation of this study is geography. Further research with samples across a range of locations would be informative. Second, there is a risk of selection bias, and efforts to capture a more representative sample would improve the reliability; however, the alignment of our findings with larger Ofcom surveys [4] suggests that this study was broadly in line with the existing literature. The survey design was pragmatic. Furthermore, survey codesign with patient and caregiver involvement could improve the uptake and validity.

## Appendix

Multimedia Appendix 1 Survey.

## Abbreviations

MATS: memory assessment and treatment service
NHS: National Health Service
UK: United Kingdom
