# Supplementary material for: Digital Access in Working-Age and Older Adults and Their Caregivers Attending Psychiatry Outpatient Clinics: Quantitative Survey
Source: JMIR Aging. 2018 Nov 22;1(2):e4. doi: 10.2196/aging.9130 (PMC6715401; doi:10.2196/aging.9130)
Supplement: Multimedia Appendix 1 [file aging_v1i2e4_app1.pdf]

Callum Cruickshank

Technology use in out patient clinics

Please take a few minutes to fill out this survey on the types of technology you have access to and what you use it for. This will help us work out what we can do to improve your care. Your answers are anonymous and will be kept confidential. Thank you for your participation.

Use of technology

Do you, or does anyone in your household, have of any of the following devices? Please tick all that apply and then indicate how often you use them

	Daily	A couple of times a week	A couple of times a month	Rarely	Never
☐ PC or laptop	☐	☐	☐	☐	☐
☐ Mobile phone	☐	☐	☐	☐	☐
☐ Tablet	☐	☐	☐	☐	☐

Do you have access to the internet?

☐ Yes | ☐ No

If yes, do you have an internet connection at home?

☐ Yes | ☐ No

Do you access the internet anywhere else? Please tick all that apply

On my mobile device ☐ Work ☐ Other: please specify

If yes, how confident do you feel accessing the internet?

☐ Not at all ☐ A little confident ☐ Moderately☐☐ Quite confident ☐ Very confident

Do you personally own a mobile phone?

☐ Yes | ☐ No

If yes, how do you connect to the internet? Please tick all that apply

☐ Wi-fi ☐ Mobile data (3G/4G) on a monthly contract

☐ It can't connect ☐ Mobile data (3G/4G) Pay as you go

Have you heard of any of the following services? Please tick all that apply

☐ Living it up ☐ Jointly ☐ Click Go

Personal Information

This will help us tailor any services we may offer in the future to the right people

What is your current age?

☐18-24 ☐25-34 ☐35-44 ☐45-54 ☐55-64 ☐65-74 ☐75+

What is your gender or sex?

☐ Male ☐ Female ☐ Other: please specify

Who is the appointment you are attending for?

☐ Yourself ☐ Someone you are accompanying

If you are accompanying someone, what is your relation to them?

☐ Son/Daughter ☐ Husband/Wife ☐ Brother/sister

☐ Friend ☐ Other: please specify

Additional Feedback

If there's anything you'd like to say about the use of technology in care, please do so here.

Thank you for taking the time to fill out our survey. We rely on your feedback to help us improve our services. Your input is greatly appreciated.
